# Transcriptomic Identification of Long Noncoding RNAs Modulating MPK3/MPK6-Centered Immune Networks in Arabidopsis

**DOI:** 10.3390/ijms26178331

**Published:** 2025-08-28

**Authors:** Tianjiao Wang, Kaifeng Zheng, Qinyue Min, Yihao Li, Xiuhua Xue, Wanjie Li, Shengcheng Han

**Affiliations:** 1 Beijing Key Laboratory of Gene Resources and Molecular Development, College of Life Sciences, Beijing Normal University, Beijing 100875, China; 202331200007@mail.bnu.edu.cn (T.W.); kaifeng_zheng@mail.bnu.edu.cn (K.Z.); xiuhuaxue@bnu.edu.cn (X.X.); lwj@bnu.edu.cn (W.L.); 2 College of Life Sciences, Qinghai Normal University, Xining 810008, China; m1nq1nyue@163.com; 3 Faculty of Arts and Sciences, Beijing Normal University, Zhuhai 519087, China; 4 Academy of Plateau Science and Sustainability of the People’s Government of Qinghai Province & Beijing Normal University, Qinghai Normal University, Xining 810008, China

**Keywords:** long noncoding RNA, plant immunity, MPK3/MPK6, pattern-triggered immunity, effector-triggered immunity

## Abstract

Mitogen-activated protein kinases 3 and 6 (MPK3/MPK6) are central to pattern-triggered immunity (PTI) and effector-triggered immunity (ETI) in Arabidopsis, yet the involvement of long noncoding RNAs (lncRNAs, >200 nt) in these pathways is poorly understood. Here, transcriptomic analyses were performed to compare lncRNA and protein-coding gene (PCG) expression profiles in wild-type (WT) and MPK3/MPK6-deficient (*MPK6SR*) Arabidopsis plants. These plants were inoculated with either *Pseudomonas syringae* pv. *tomato* (*Pst*) DC3000, which elicits both PTI and ETI, or its type III secretion-deficient mutant, *Pst* DC3000 *hrcC*^−^, which induces only PTI. RNA sequencing (RNA-seq) analysis of 18 samples identified 1388 known and 70 novel lncRNAs, among which differentially expressed lncRNAs (DElncRNAs) involved in disease resistance were further identified. Using integrative analyses, including weighted gene co-expression network analysis (WGCNA), prediction of lncRNA *cis*-regulatory targets for PCGs, and validation via reverse transcription-quantitative PCR (RT-qPCR), three core lncRNA-mediated regulatory modules were identified: (i) MPK3/MPK6-dependent PTI and ETI, where lncRNAs amplify signals; (ii) MPK3/MPK6-dependent PTI, where lncRNAs fine-tune basal immunity; and (iii) MPK3/MPK6-independent PTI and ETI, where lncRNAs serve as a backup regulatory network. These modules form a multi-layered immune regulatory network via *cis*- and *trans*-regulation and further enable the identification of lncRNA-PCG pairs involved in both regulatory modes. This work enhances the understanding of the molecular mechanisms underlying plant innate immunity.

## 1. Introduction

The long-term coevolution between plants and pathogens has sculpted a complex, integrated set of innate immune mechanisms. Unlike animals, which rely on mobile immune cells and adaptive immunity, plants depend on cell-autonomous defense mechanisms and systemic signaling that originates at infection sites [1]. PTI is initiated when membrane-localized pattern recognition receptors (PRRs) specifically recognize evolutionarily conserved pathogen/microbe-associated molecular patterns (PAMPs/MAMPs), providing basal disease resistance. Intracellularly, pathogen effectors are detected by nucleotide-binding leucine-rich repeat (NLR) receptors, which elicit a second defensive layer, ETI. ETI confers more robust and prolonged defense responses. Together, PTI and ETI act synergistically to bolster disease resistance and initiate downstream defense response cascades [2]. Both pathways involve mitogen-activated protein kinase (MAPK) cascades, with MPK3 and MPK6 serving as central regulators of downstream immune signaling [3]. During PTI, PRR activation triggers the sequential phosphorylation of MITOGEN-ACTIVATED PROTEIN KINASE KINASE KINASEs (MAPKKKs, such as MEKK1), followed by MITOGEN-ACTIVATED PROTEIN KINASE KINASEs (MAPKKs, such as MKK4/5), and ultimately MPK3 and MPK6. Once activated, these kinases phosphorylate downstream target transcription factors, thereby inducing the transcriptional activation of defense-related genes [4]. ETI typically induces a hypersensitive response (HR)—a localized programmed cell death process that restricts pathogen spread—and also amplifies PTI-associated signaling cascades. Moreover, MPK3 and MPK6 are activated during ETI, with their activation exhibiting greater amplitude and longer duration than in PTI. This differential activation profile underscores the essential regulatory role of these kinases in both tiers of plant innate immunity [5,6].

LncRNAs, defined as transcripts exceeding 200 nucleotides without protein-coding capacity, are ubiquitous elements in plant genomes [7,8]. Although they are characterized by lower abundance, diminished sequence conservation, and heightened tissue-specificity compared with PCGs [9,10,11], lncRNAs critically regulate plant development, growth, and environmental adaptation [12]. Their functional mechanisms include chromatin remodeling, transcriptional modulation, and miRNA sequestration [13,14,15]. Primarily transcribed by RNA polymerase II (with minor contributions from other polymerases), lncRNAs are classified by genomic location into major categories: long intergenic noncoding RNAs (lincRNAs), intronic lncRNAs (incRNAs), natural antisense transcripts (NATs), and sense lncRNA [16]. Across diverse plant species, lncRNAs are increasingly recognized as pivotal regulators of biotic stress responses. In *Arabidopsis thaliana* (*A. thaliana*), the lincRNA ELF18-INDUCED LONG NONCODING RNA1 (ELENA1) interacts with MEDIATOR SUBUNIT 19A (MED19a) to upregulate PATHOGENESIS-RELATED GENE1 (PR1), enhancing resistance to *Pseudomonas syringae* [17]; while *Fusarium oxysporum*-responsive lincRNAs confer disease resistance through uncharacterized mechanisms, evidenced by exacerbated symptoms upon their depletion [18]. In tomato (*Solanum lycopersicum*), a NAT-lncRNA bolsters late blight resistance by inducing the expression of its cognate gene *SIGRX21* [19], whereas silencing of the lincRNAs slylnc0195 and slylnc1077 significantly elevates *Tomato Yellow Leaf Curl Virus* (TYLCV) accumulation [20]. LncRNAs in *Nicotiana attenuata*, especially early-responding lincRNAs such as JAL1 and JAL3, regulate JA-mediated herbivore defenses by affecting JA levels, related defenses, and resistance, while late-responding lincRNAs are transcriptionally regulated by JA signaling [21]. Rice (*Oryza sativa*) lncRNA *ALEX1* interacts with transcription factors to activate defense genes against *Magnaporthe oryzae* [22]. Functional analysis of potato lincRNAs identified 17 candidates co-expressed with defense genes during *Pectobacterium carotovorum* infection [23]. Wheat lincRNAs further contribute to biotic stress regulation during stripe rust and powdery mildew challenges [24]. Collectively, these studies highlight the essential regulatory roles of lncRNAs in plant biotic stress responses.

The precise regulatory mechanisms of lncRNAs in MPK3/MPK6-centered immune networks, particularly the coordination of their *cis*- and *trans*-regulatory modalities across PTI and ETI pathways, remain insufficiently characterized [25]. To resolve this knowledge gap, transcriptome profiling of lncRNAs and PCGs in *A. thaliana* WT and *MPK6SR* mutants challenged with *Pst* DC3000 or *Pst* DC3000 *hrcC^−^* was performed. By integrating WGCNA, *cis*-regulatory target prediction, and RT-qPCR validation, the regulatory elements mediated by lncRNAs in both MPK3/MPK6-dependent and -independent immune pathways were delineated. The analysis identified 1388 annotated and 70 novel lncRNAs with distinct genomic architectures. WGCNA further identified three core modules: (i) MPK3/MPK6-dependent PTI and ETI, (ii) MPK3/MPK6-dependent PTI, and (iii) MPK3/MPK6-independent PTI and ETI, with lncRNAs exerting dual *cis/trans* regulatory functions. LncRNAs in the three core modules may function through different regulatory methods: in MPK3/MPK6-dependent PTI and ETI, lncRNAs amplify immune signals by targeting MAPK cascade components and downstream defense genes, enhancing pathogen recognition and response intensity; in MPK3/MPK6-dependent PTI specifically, they regulate basal immunity via metabolic processes and membrane signaling to support rapid defense against non-virulent pathogens; in MPK3/MPK6-independent PTI and ETI, they act as a “backup network” through the regulation of cytoplasmic transcription factors and vesicle transport proteins, maintaining immune homeostasis even when the core MAPK cascade is impaired. This work collectively establishes lncRNAs as versatile coordinators of MPK3/MPK6-centered immune networks, advancing mechanistic understanding of plant immunity and enabling future dissection of immune regulatory complexity.

## 2. Results

### 2.1. Characterization of Pathogen-Induced Immune Responses and Genome-Wide Identification of lncRNAs in Arabidopsis

Four-week-old soil-grown *A. thaliana* WT and NA-PP1-treated *MPK6SR* mutants were inoculated with *Pst* DC3000 or *Pst* DC3000 *hrcC*^−^ suspensions (4 × 10^8^ CFU/mL) for 1 h. The *MPK6SR* mutant is a conditional loss-of-function allele generated through site-specific modification of the MPK6 locus and exhibits abrogated MPK3/MPK6 activity when treated with NA-PP1. This line was selected for the present study based on the well-established role of MPK3 and MPK6 as core MAPKs in plant immune signaling, whereas their regulatory crosstalk with lncRNAs in mediating disease resistance remains to be elucidated. For bacterial inoculation, *Pst* DC3000 triggers both PTI and ETI via a functional type III secretion system that mediates effector translocation. In contrast, the *hrcC^−^* mutant—defective in type III secretion—induces only PTI. This pair of strains allowed the dissection of lncRNA functions in distinct branches of immune signaling. The expression levels of the PTI marker gene *FLG22-INDUCED RECEPTOR-LIKE KINASE 1 (FRK1)* and ETI marker gene *BONZAI 3 (BON3)* in WT plants were quantified via RT-qPCR [26]. Both *Pst* DC3000 and *Pst* DC3000 *hrcC*^−^ significantly induced these genes within 1 h compared with mock controls, confirming successful pathogen establishment (Figure S1). In *MPK6SR* mutants, *Pst* DC3000 challenge elevated *FRK1* and *BON3* expression relative to mock controls. By contrast, *Pst* DC3000 *hrcC*^−^ inoculation only upregulated *BON3* without altering *FRK1* levels, revealing differential MPK3/MPK6 pathway activation in response to contrasting virulence effectors (Figure S1).

**Figure 1:**
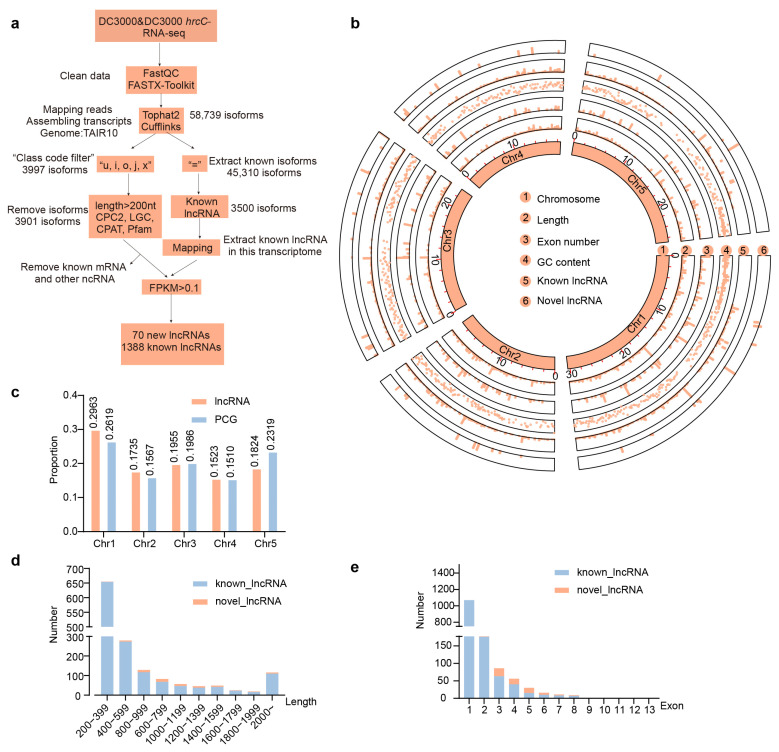
Genome-wide identification and feature analysis of lncRNAs in *A. thaliana*. (**a**) Workflow for lncRNA identification in *A. thaliana*. (**b**) Genome-wide features of *A. thaliana* lncRNAs. Circles 1–4 display the chromosomal distribution of various attributes of all lncRNAs. Circles 5 and 6 show the chromosomal localization of known and newly identified lncRNAs, respectively. (**c**) Chromosomal proportion of lncRNAs versus PCGs in *A. thaliana*. (**d**) Length variation between novel and known lncRNAs in *A. thaliana*. (**e**) Exon count differences between novel and known lncRNAs in *A. thaliana*.

RNA-seq at a sequencing depth of 10 GB generated 18 datasets for lncRNA identification. Prior to transcriptome assembly, rigorous quality control was performed on the sequencing data. To ensure data reliability, key bioinformatics quality metrics were evaluated, including high read alignment rates, rRNA contamination percentages, and sequence duplication rates (Table S1). Transcriptome assembly against the TAIR10 reference genome produced 58,739 isoforms. Applying an integrated screening pipeline—incorporating transcript class code, length filtering (>200 nt), coding potential assessment (CPC2/LGC/CPAT/Pfam), known RNA exclusion, and FPKM threshold >0.1—1388 annotated lncRNAs and 70 novel lncRNAs (all class code “j”) were identified (Figure 1a, Tables S2 and S3). To assess the conservation of the 70 novel lncRNAs identified in this study, their phylogenetic distribution was analyzed, and homologous sequence alignment was performed against lncRNA sequences from 39 distinct species. A total of 17 homologous lncRNAs were detected, and all of these were restricted to eudicot plants (e.g., *Arabidopsis lyrata*, *Brassica rapa*, and *Brassica napus*). The functional annotations of these lncRNAs—which have homologous sequences in other species—were further queried, and none were found to have been characterized in previous studies (Figure S2, Table S4).

Chromosomal distribution analysis and characterization of fundamental features revealed that lncRNAs are distributed across all five chromosomes, with a significant enrichment on chromosome 1 (29.63%) and underrepresentation on chromosome 5 (18.24%) (Figure 1b,c). Length distribution profiling indicated predominant clustering of lncRNAs within 200–399 bp, while novel lncRNAs demonstrated expanded representation in the 600–1200 bp range (Figure 1d, Table S5). Exon architecture analysis showed that most lncRNAs contained 1–3 exons, though novel variants exhibited increased exon counts (up to 8) (Figure 1e, Table S6). These results collectively delineate the architectural landscape of pathogen-responsive lncRNAs in *A. thaliana.*

### 2.2. Differential Expression Analysis of lncRNAs and PCGs in Response to Pathogen Infection

**Figure 2:**
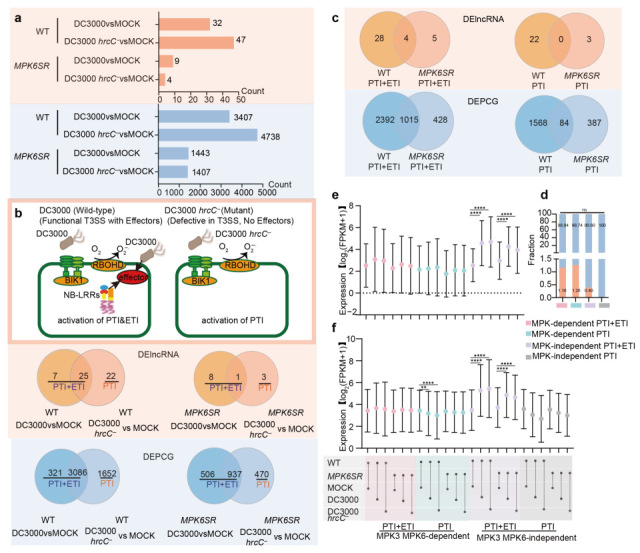
Transcriptional reprogramming of lncRNAs and PCGs during pathogen challenge. (**a**) The number of DElncRNAs/DEPCGs across genotypes (WT vs. *MPK6SR*) under different infection conditions (|log_2_FC| ≥ 1, *p* value ≤ 0.05). (**b**) Schematic of plant immunity activation by *Pst* DC3000 and *Pst* DC3000 *hrcC^−^*, along with counts of DElncRNAs and DEPCGs under distinct pathogenic stimulations in WT and *MPK6SR* plants. (**c**) Intersectional relationships of MPK3/6-dependent and -independent transcript sets. (**d**) Proportional representation of DElncRNAs to DEPCGs in immune-related pathways. (ns: *p* > 0.05, Fisher’s exact test). (**e**,**f**) Comparative expression magnitude analysis of DElncRNAs and DEPCGs. Expression levels in each sample are computed in log_2_ (FPKM + 1) units. (**: *p* < 0.01, ****: *p* < 0.0001, Mann–Whitney *U* test).

Differential expression analysis was performed across six experimental conditions to characterize the dynamics of lncRNAs and PCGs in response to immune activation by *Pst* DC3000 and *Pst* DC3000 *hrcC^−^* in both WT and *MPK6SR* backgrounds. Specifically, the following comparisons were conducted: *Pst* DC3000 (1 hpi vs. 0 hpi) and *Pst* DC3000 *hrcC^−^* (1 hpi vs. 0 hpi). Transcripts showing significant differential expression (|log_2_FC| ≥ 1, *p* value ≤ 0.05) were categorized as DElncRNAs or differentially expressed PCGs (DEPCGs) (Figure 2a, Table S7). Wild-type plants exhibited 32 DElncRNAs and 3407 DEPCGs following 1 h *Pst* DC3000 challenge, alongside 47 DElncRNAs and 4738 DEPCGs under *Pst* DC3000 *hrcC^−^* treatment. By contrast, *MPK6SR* mutants showed markedly attenuated responses, with only nine DElncRNAs/1443 DEPCGs (*Pst* DC3000) and four DElncRNAs/1407 DEPCGs (*Pst* DC3000 *hrcC^−^*) (Figure 2a).

*Pst* DC3000 elicits dual immune responses (PTI and ETI) in *A. thaliana*, whereas its type III secretion system-deficient mutant *Pst* DC3000 *hrcC^−^* exclusively activates PTI. Wild-type plants exhibited 32 DElncRNAs and 3407 DEPCGs involved in PTI and ETI, compared with 22 DElncRNAs and 1652 DEPCGs involved in PTI. The corresponding *MPK6SR* mutant responses showed attenuated profiles: nine DElncRNAs/1443 DEPCGs (PTI and ETI) and three DElncRNAs/470 DEPCGs (PTI) (Figure 2b). The *MPK6SR* chemogenetic mutant, featuring chemically induced MPK3/MPK6 double knockout, enabled delineation of MPK3/MPK6-dependent regulation. Screening PTI and ETI responsive DElncRNAs revealed four overlapping transcripts functioning independently of MPK3/MPK6 (designated MPK3/MPK6-independent DElncRNAs), and thirty-three unique MPK3/MPK6-dependent DElncRNAs. Parallel analyses identified 1015 MPK3/MPK6-independent and 2820 dependent DEPCGs. For PTI responses, screening analyses detected no overlapping DElncRNAs independent of MPK3/MPK6 but identified 25 DElncRNAs dependent on MPK3/MPK6. Corresponding to these two categories, the counts of DEPCGs were 84 (for the MPK3/MPK6-independent group) and 1955 (for the MPK3/MPK6-dependent group), respectively (Figure 2c, Table S8).

Further investigation into how pathogen resistance signals regulate differentially expressed genes (DEGs) revealed conserved DElncRNA/DEPCG abundance ratios across immune pathways (Figure 2d), indicating that lncRNA-mediated PCG regulation operates independently of total gene number variations. While DElncRNA expression showed no significant differences between MPK3/MPK6-dependent pathways, DEPCGs in *Pst* DC3000 *hrcC^−^*-induced PTI exhibited marked downregulation in wild-type plants post-stimulation. This suppression, unlinked to lncRNA regulation, implies modulation by complex lncRNA-independent mechanisms. Notably, within MPK3/MPK6-independent PTI and ETI pathways, coordinated induction of both DElncRNAs and DEPCGs occurred in *Pst* DC3000- or *Pst* DC3000 *hrcC^−^*-infected plants, suggesting potential lncRNA-mediated transcriptional control (Figure 2e,f).

Collectively, differential expression analysis delineates distinct responsiveness of lncRNAs and PCGs to pathogen infection signals while establishing the MPK3/MPK6 signaling pathway as a central modulator of these transcriptional dynamics. These findings elucidate regulatory hierarchies within plant immune networks and enable molecular dissection of pathogen resistance mechanisms.

### 2.3. WGCNA Identification of Core Modules and Functional Analysis of lncRNA-PCG Regulatory Networks

**Figure 3:**
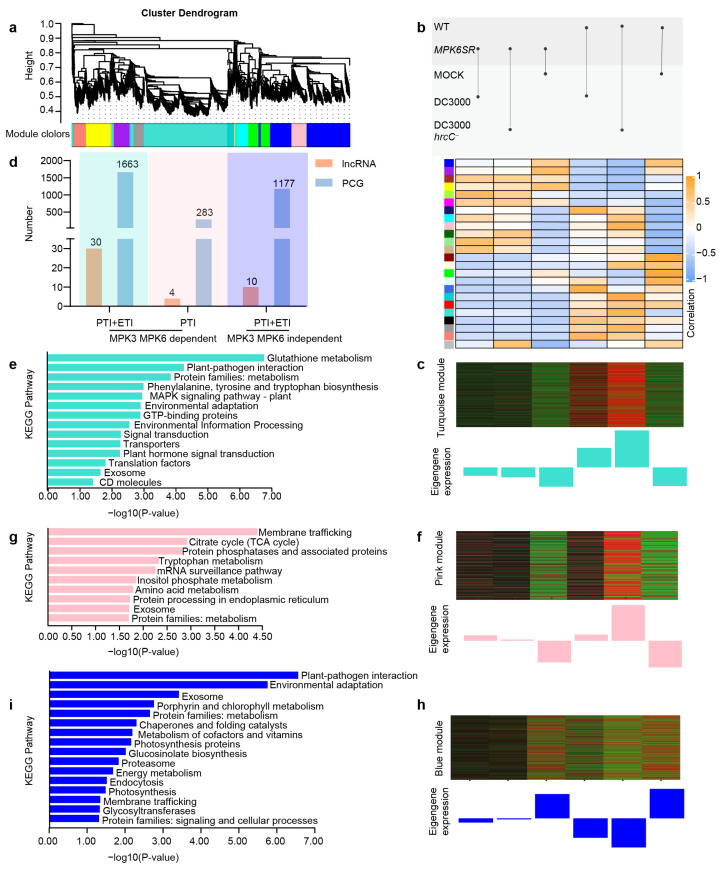
Co-expression network analysis of *trans*-targeting lncRNA-PCG interactions in *A. thaliana* immunity. (**a**) Hierarchical clustering dendrogram with module assignment (24 co-expression modules; *n* = 8000 transcripts). (**b**) Module-trait correlation heatmap. (**c**) “Turquoise” module eigengene dynamics across genotypes (WT vs. *MPK6SR*) under *Pst* DC3000 and *Pst* DC3000 *hrcC^−^* challenges. (**d**) Module-wise distribution of lncRNA and protein-coding gene counts. (**e**) KEGG pathway enrichment for “turquoise” module *trans*-regulated lncRNA-PCG pairs. (**f**) “Pink” module eigengene expression patterns in plant–pathogen interactions. (**g**) Significantly enriched KEGG terms among “pink” module *trans*-targeting networks. (**h**) “Blue” module eigengene trajectories during immune responses. (**i**) Functional annotation of “blue” module *cis*-regulatory associations via KEGG.

WGCNA was performed on 1458 lncRNAs and 32,185 PCGs to elucidate *trans*-regulatory mechanisms in seedling immune pathways. Following quality control, hierarchical clustering of 8000 filtered genes generated 24 co-expression modules through dendrogram branch segregation (Figure 3a). Co-expression patterns of module-embedded PCGs permit functional annotation of sparsely represented lncRNAs. Correlation assessments against immune treatments identified three key modules—turquoise, pink, and blue—as being strongly correlated with specific immune regulatory processes (Figure 3b, Table S9).

**Figure 4:**
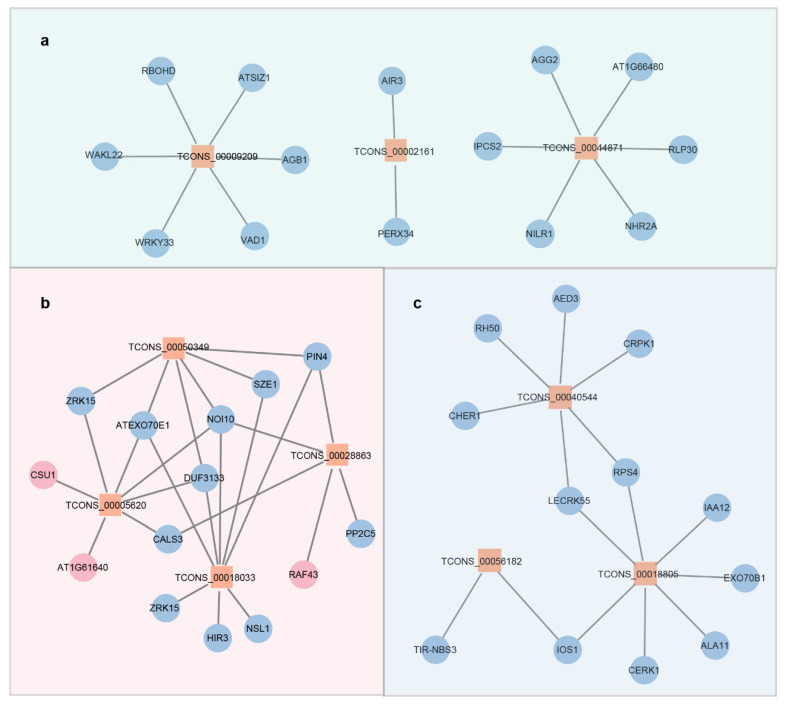
Transcriptomic co-regulatory networks linking lncRNAs with *trans*-targeted PCGs. (**a**) Network architecture of the “turquoise” module (edge weight ≥ 0.4). (**b**) Interactome mapping for the “pink” module (edge weight ≥ 0.4). (**c**) Regulatory connectivity within the “blue” module (edge weight ≥ 0.4).

Transcripts within the turquoise module exhibited pronounced upregulation in wild-type seedlings challenged with *Pst* DC3000 or *Pst* DC3000 *hrcC^−^*, while remaining unresponsive to other treatments (Figure 3c). Comprising 30 lncRNAs and 1663 PCGs (Figure 3d), this module is functionally associated with MPK3/MPK6-dependent PTI and ETI signaling. KEGG enrichment analysis revealed broad pathway representation among module PCGs, encompassing metabolism (glutathione/amino acid metabolism), signal transduction (MAPK/phytohormone signaling), and environmental adaptation (plant–pathogen interactions), collectively revealing the complex molecular network activated during dual PTI-ETI induction (Figure 3e). Within the turquoise module, three *trans*-regulatory hub lncRNAs (TCONS_0009209, TCONS_00002161, and TCONS_00044871) were identified, each forming decentralized hub-and-spoke networks through interactions with five PCGs on average, reflecting multi-pathway synergy during dual immune activation (Figure 4a). Functional diversification is evidenced by their distinct target associations: TCONS_0009209 co-expresses with *RESPIRATORY BURST OXIDASE PROTEIN D (RBOHD)* and *WRKY TRANSCRIPTION FACTOR 33 (WRKY33)*; TCONS_00002161 interacts with the cell wall remodeling genes *AUXIN-INDUCED IN ROOT CULTURES 3 (AIR3)* and *PEROXIDASE 34 (PERX34)*; while TCONS_00044871 connects to *G-PROTEIN GAMMA SUBUNIT 2 (AGG2)* and disease resistance gene *NEMATODE-INDUCED LRR-RLK 1 (NILR1)*, collectively mediating specialized immune functions (Figure 4a).

The “pink” module is mainly expressed in wild-type seedlings treated with *Pst* DC3000 *hrcC^−^*, and its transcript levels are barely affected by other treatments (Figure 3f). Composed of 4 lncRNAs and 283 PCGs (Figure 3d), this module is hypothesized to be involved in “MPK3/MPK6-dependent PTI”. KEGG enrichment analysis indicated that relatively few pathways are enriched in this module, which are mainly focused on metabolic processes (such as the TCA cycle and tryptophan metabolism), protein processing (protein processing in the endoplasmic reticulum), and membrane trafficking (Figure 3g). Within the “pink” module, four lncRNAs (TCONS_00050349, TCONS_00028863, TCONS_00005620, and TCONS_00018033) form a tightly interconnected network that regulates basal immune responses (Figure 4b). For instance, the auxin transport gene *PIN-FORMED 4 (PIN4)* is co-expressed with TCONS_00050349; *CALLOSE SYNTHASE 3 (CALS3)* is associated with TCONS_00005620; and *RAF-LIKE MITOGEN-ACTIVATED PROTEIN KINASE KINASE KINASE 43 (RAF43)* and *PHOSPHATASE 2C5 (PP2C5)* are connected to TCONS_00028863 (Figure 4b). These interactions suggest that the “pink” module may act as a key regulatory hub in the basal immune response of WT seedlings to *Pst* DC3000 *hrcC^−^* treatment.

The “blue” module shows primary expression in mock-treated WT and *MPK6SR* seedlings, with its transcript levels downregulated by other treatments (Figure 3h). Comprising 10 lncRNAs and 1177 PCGs (Figure 3d), this module is proposed to be involved in “MPK3/MPK6-independent PTI and ETI”. KEGG enrichment analysis identified a moderate number of pathways within this module, with notable emphasis on “innate basic pathways” such as plant–pathogen interaction and environmental adaptation, along with photosynthesis and vesicle transport (Figure 3i). Within the “blue” module, three lncRNAs (TCONS_00040544, TCONS_00056182, and TCONS_00018805) form a “multi-center interconnection network” together with PCGs (Figure 4c). For example, bacterial response molecules, including *COLD-RESPONSIVE PROTEIN KINASE 1 (CRPK1)*, disease resistance gene *RESISTANT TO P. SYRINGAE 4 (RPS4)*, and *LECTIN RECEPTOR KINASE 55 (LECRK55)*, are associated with TCONS_00040544; antidisease gene *TOLL/INTERLEUKIN RECEPTOR-NUCLEOTIDE BINDING SITE 3 (TIR-NBS3)* and plant pathogen-responsive receptor-like kinase gene *IMPAIRED OOMYCETE SUSCEPTIBILITY 1 (IOS1)* are associated with TCONS_00056182; and auxin signaling gene *INDOLE-3-ACETIC-ACID INDUCIBLE 12 (IAA12)* and vesicle transport-related gene *EXOCYST SUBUNIT EXO70 FAMILY PROTEIN B1 (EXO70B1)* are connected to TCONS_00018805 (Figure 4c). These findings collectively suggest that the “blue” module plays a distinct regulatory role in plant immune responses independent of MPK3/MPK6.

### 2.4. Cis-Regulatory lncRNA-PCG Pairs and Functional Classification of Proteins in Immune Pathways

**Figure 5:**
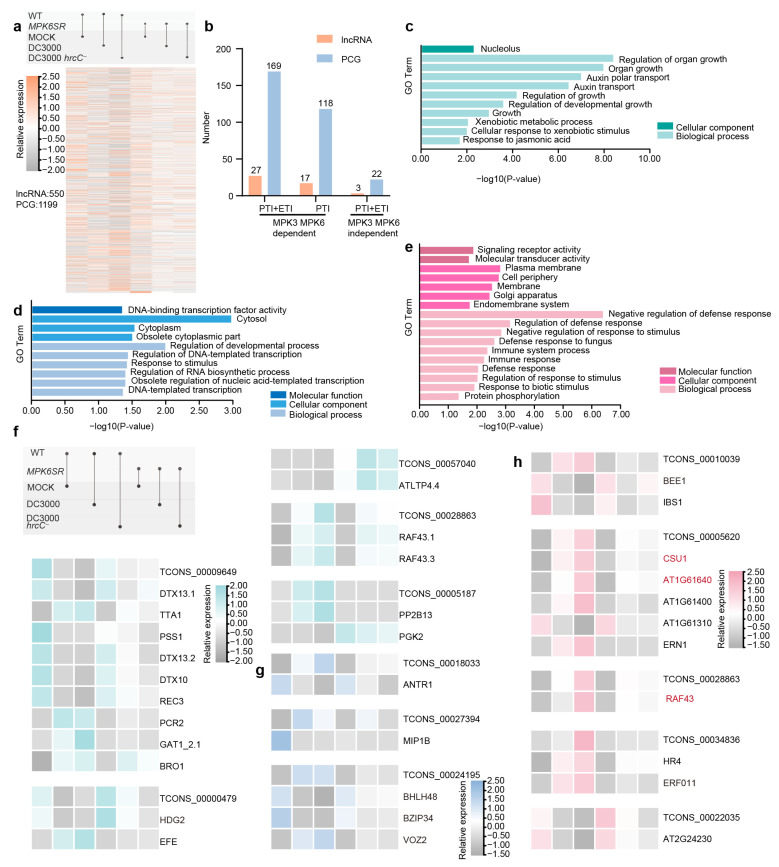
*Cis*–regulatory lncRNA–PCG pairs and functional classification of proteins in Arabidopsis immune pathways. (**a**) Heatmap of co-expressed lncRNA-PCG pairs. (**b**) Number of *cis*-regulatory lncRNAs and *cis*-targeted PCGs in different immune pathways. (**c**) GO enrichment analysis for MPK3/MPK6-dependent PTI and ETI pathways. (**d**) GO enrichment analysis for the MPK3/MPK6-independent PTI and ETI pathways. (**e**) GO enrichment analysis for the MPK3/MPK6-dependent PTI pathway. (**f**) Heatmap of functional classification of proteins in the MPK3/MPK6-dependent PTI and ETI pathways. (**g**) Heatmap of functional classification of proteins in the MPK3/MPK6-independent PTI and ETI pathways. (**h**) Heatmap of functional classification of proteins in the MPK3/MPK6-dependent PTI pathway. (Log scale: base = 2; log width = 1; row scale: normalized).

Employing Bedtools software and using the *A. thaliana* TAIR10 database as the reference genome, potential *cis*-regulatory target PCGs were identified by targeting a 100-kilobase (kb) window upstream and downstream of selected lncRNA transcription sites, aiming to explore PCGs potentially subject to *cis*-regulation by these lncRNAs. To further screen lncRNA-PCG pairs with significant co-expression, Spearman correlation coefficients for all pairs were computed and strict thresholds (|ρ| ≥ 0.9 and *p* < 0.01) applied, identifying 1241 qualifying pairs involving 550 lncRNAs and 1199 PCGs (Figure 5a, Table S10). These pairs were further categorized by plant immune regulatory pathways: 184 pairs (27 lncRNAs, 169 PCGs) in the MPK3/MPK6-dependent PTI and ETI pathways; 861 pairs (17 lncRNAs, 118 PCGs) in the MPK3/MPK6-dependent PTI pathway; and 22 pairs (3 lncRNAs, 22 PCGs) in the MPK3/MPK6-independent PTI and ETI pathways (Figure 5b, Table S11).

To explore the role of lncRNAs in plant immunity, Gene Ontology (GO) enrichment analysis was performed across different pathways, revealing distinct functional features associated with their varying involvement. In the MPK3/MPK6-dependent PTI and ETI pathways, only the nucleolus was significantly enriched at the cellular component level; biological processes mainly involved organ growth regulation and auxin polar transport, reflecting the deep integration of growth and immune signals, which coordinate immune responses by influencing cell division and differentiation (Figure 5c). Further analysis of the “turquoise” module, associated with this pathway, highlighted key genes such as the plant disease resistance-related gene *ETHYLENE-FORMING ENZYME (EFE)*, the growth-related gene *TITANIA 1 (TTA1)*, and the homeodomain transcription factor gene *HOMEODOMAIN GLABROUS 2 (HDG2)*. Notably, the lncRNA (TCONS_00000479) may exert its function by regulating *HDG2*, implying the module’s role in balancing growth and disease resistance (Figure 5f).

In the MPK3/MPK6-dependent PTI pathway, molecular functions focused on signal receptor and transduction activity, with cellular components enriched in the plasma membrane and Golgi apparatus; biological processes primarily involved fungal defense and immune responses, indicating a reliance on membrane signal transduction for rapid pathogen defense (Figure 5e). Correspondingly, the “pink” module associated with this pathway showed that the lncRNAs TCONS_00010039 and TCONS_00034836 regulate transcription factor genes *BR ENHANCED EXPRESSION 1 (BEE1)* and *ETHYLENE RESPONSIVE FACTOR 011 (ERF011)*, respectively, to mediate these processes. Among these genes, *COP1 SUPPRESSOR 1 (CSU1)*, *AT1G61640,* and *RAF43* were identified in both *trans*- and *cis*-regulatory analyses, suggesting a high potential for involvement in lncRNA-mediated regulation; their expression levels will be validated by RT-qPCR in subsequent studies (Figure 5h).

In the MPK3/MPK6-independent PTI and ETI pathways, core molecular functions involved DNA-binding transcription factor activity, with cellular components concentrated in the cytoplasm; biological processes included developmental regulation and DNA template transcription, suggesting a mechanism that bypasses MPK3/MPK6 to link immunity with long-term growth balance via cytoplasmic transcription factors (Figure 5d). In the “blue” module corresponding to this pathway, transcription factor genes *BASIC HELIX-LOOP-HELIX 48 (BHLH48)*, *BASIC REGION/LEUCINE ZIPPER MOTIF 34 (BZIP34)*, and *VASCULAR PLANT ONE ZINC FINGER PROTEIN 2 (VOZ2)* were detected, which may be regulated by the lncRNA TCONS_00024195 (Figure 5g).

### 2.5. Validation of RNA-Seq Data by RT-qPCR Analysis

**Figure 6:**
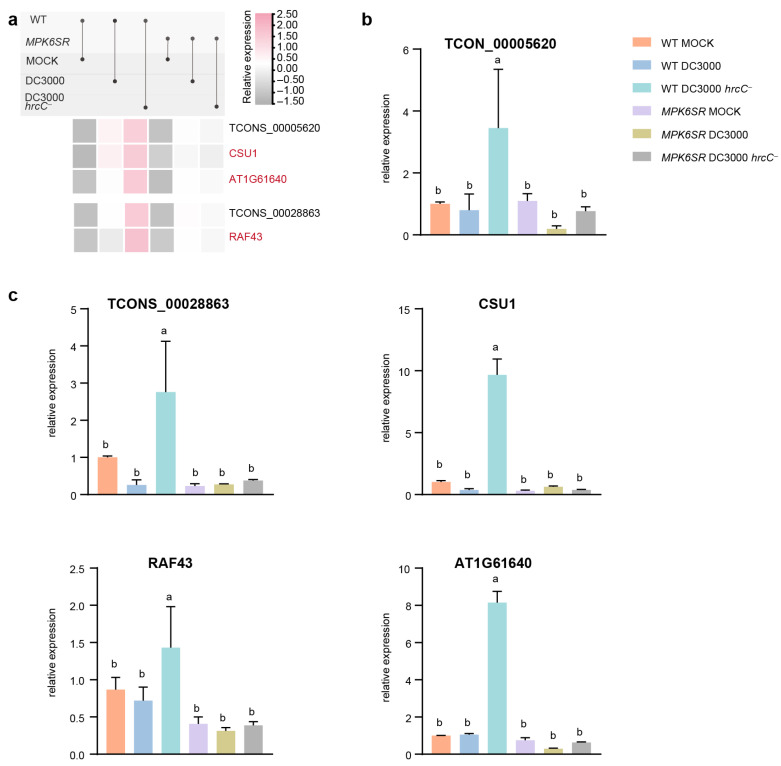
RT-qPCR validation of RNA-seq data for lncRNA-PCG pairs in the Arabidopsis immune response. (**a**) Heatmap of RNA-seq Data for lncRNA-PCG pairs. (**b**) RT-qPCR validation of lncRNA TCONS_00028863 and its target gene *RAF43*. (**c**) RT-qPCR validation of lncRNA TCONS_00005620 and its target genes *AT1G61640* and *CSU1*. Error bars represent the standard error of triplicate experiments, and statistical significance with *p* < 0.05 was checked by a two-way ANOVA. This can explain a and b.

To verify the reliability of the RNA-seq results (Figure 6a), RT-qPCR was performed on selected lncRNAs and their target PCGs, which had been previously identified as both *trans*- and *cis*-regulated targets. The analyzed molecules included lncRNA TCONS_00028863 and its target gene *RAF43* (Figure 6b), as well as lncRNA TCONS_00005620 and its target PCGs *AT1G61640* and *CSU1* (Figure 6c). The RT-qPCR-based transcript profiling largely confirmed the RNA-seq data: in WT treated with the *Pst* DC3000 *hrcC^−^* pathogen, the expression levels of these genes were significantly elevated compared with other treatment groups, consistent with the expression pattern of the “MPK3/MPK6-dependent PTI” pathway.

## 3. Discussion

Plant resistance to bacterial pathogens depends on coordinated immune signaling networks. In this study, the functionality of lncRNAs in *A. thaliana* responding to virulent *Pst* DC3000 and its type III secretion system-deficient mutant *Pst* DC3000 *hrcC^−^* was delineated, with a specific focus on the integration of lncRNA regulation with MPK3/MPK6 signaling. A total of 61 disease resistance-associated lncRNAs were identified (Table S12). A comprehensive search of the prior literature revealed 88 previously reported immune-related lncRNAs in *A. thaliana* [17,27,28,29]. Subsequent comparative analysis using BedTools demonstrated that none of these 61 disease resistance-associated lncRNAs had been characterized in previous studies. By integrating WGCNA, *cis*-regulatory target prediction, and RT-qPCR validation, it is established that lncRNAs critically orchestrate immune responses via both MPK3/MPK6-dependent and -independent pathway lncRNAs critically orchestrate immune responses via both MPK3/MPK6-dependent and -independent pathways, forming a layered defense architecture against pathogens.

Pathogen infection efficacy was validated by profiling the PTI marker *FRK1* and the ETI marker *BON3* (Figure S1). Wild-type plants exhibited significant upregulation of both genes upon challenge with *Pst* DC3000 (which induces both PTI and ETI) or *Pst* DC3000 *hrcC^−^* (which induces only PTI), confirming successful immune activation. NA-PP1-treated *MPK6SR* mutants were genetically deficient in MPK3 and MPK6. In these mutants, *Pst* DC3000 still induced FRK1 and BON3 expression; however, *Pst* DC3000 *hrcC^−^* only activated BON3 and failed to induce FRK1. This observation is particularly striking: MPK3 and MPK6 are well established as core PTI signaling hubs that transduce PRR-derived signals [4,30,31]. The findings refine the current understanding of plant immune signaling by uncovering pathway-specific functional divergence. Specifically, MPK3 and MPK6 are indispensable for PTI—this requirement is especially critical for defense against *Pst* DC3000 *hrcC^−^*. In contrast, ETI appears to rely on compensatory signaling cascades that bypass these kinases. This result aligns with prior studies, which highlighted key functional distinctions in MPK3/MPK6 contributions to the PTI versus ETI pathway [32].

Classification of “MPK3/MPK6-dependent” and “MPK3/MPK6-independent” DElncRNAs/DEPCGs revealed that all DElncRNAs in *Pst* DC3000 *hrcC^−^*-induced PTI are MPK3/MPK6-dependent, whereas a subset of independent lncRNAs persists in *Pst* DC3000-induced PTI and ETI. This observation suggests a more critical role for MPK3/MPK6 in lncRNA regulation under single PTI activation and potential compensatory mechanisms under synergistic PTI and ETI. While the ratio of DElncRNAs to DEPCGs showed no significant pathway-specific differences, expression changes indicated both lncRNA-dependent and -independent PCG regulation—implying plants fine-tune PCG expression balance via lncRNA-mediated control combined with other complex networks (Figure 2). This aligns with reports of lncRNAs engaging in intricate regulatory networks in plants [33,34]. The coexistence of these two regulatory modes likely reflects evolutionary adaptation to pathogen diversity, as seen in *A. thaliana*, where *Fusarium oxysporum*-responsive lncRNAs interact with core immune components and crosstalk with metabolic pathways to ensure robust defense even when signaling nodes are inhibited [18]. This highlights lncRNAs as pivotal nodes balancing energy cost and defense efficacy.

The WGCNA analysis identified three immune pathways. The turquoise module, which is MPK3/MPK6-dependent and associated with both PTI and ETI, is enriched in glutathione metabolism, MAPK signaling, and other processes. This enrichment highlights the integration of multiple signaling layers in plant immunity: glutathione metabolism, recognized as a central hub linking redox homeostasis to immune activation, modulates ROS balance by upregulating ROS-scavenging genes (e.g., APX, CAT) and coordinates with salicylic acid (SA)-mediated signaling to enhance resistance, as demonstrated in *Nicotiana benthamiana* challenged with tobacco mosaic virus (TMV) [35]. This aligns with the observation that this module likely utilizes glutathione to fine-tune ROS levels, supporting pathogen restriction while preventing excessive oxidative damage [36]. Consistent with previous studies showing the importance of glutathione metabolism and MAPK signaling in plant immune responses [37,38,39], the findings suggest lncRNAs play a role in coordinating these processes. Strong co-expression was observed between the core lncRNA TCONS_0009209 and *WRKY33*—a known downstream PCG of MPK3/MPK6 that regulates camalexin biosynthesis [40,41]. This suggests that *WRKY33* may be enhanced by TCONS_0009209—analogous to the regulation of *PR1* by ELENA1 [17]—thereby amplifying MPK3/MPK6-mediated immune signaling. The *cis*-target gene of TCONS_00000479, *HDG2* (a class IV HD-ZIP transcription factor gene), is regulated by TCONS_00000479 (another core lncRNA) via *cis*-acting mechanisms—potentially through binding to the *HDG2* promoter, modulating chromatin accessibility, or regulating transcription initiation. These actions enable precise spatiotemporal control over *HDG2*’s expression. HDG2 is specifically localized to the epidermal layer (L1) of shoot apical meristems and floral organs, and its expression level directly determines the capacity to regulate downstream growth-related genes [42]. Collectively, these findings imply the immune–growth balance can be coordinated by lncRNAs in the turquoise module—via the amplification of immune signaling and fine-tuning of growth-related gene regulation, respectively.

The pink module, an MPK3/MPK6-dependent PTI module, is enriched in basic metabolic processes (e.g., the TCA cycle) and protein processing. Core lncRNAs (e.g., TCONS_00028863) interact with the MAPKKK gene *RAF43* and protein phosphatase 2C gene *PP2C5* to strengthen membrane signal transduction for rapid defense responses (Figure 4b), consistent with the notion that rapid PTI defense involves membrane-associated signaling [43,44,45]. Specifically, RAF43 likely acts as an upstream activator of MAPK cascades, rapidly engaged post-PRR activation, as seen in flg22-induced MPK3/MPK6 activation within 1–5 min, to amplify signals from membrane-localized PRRs to intracellular targets [46,47]. Immune signaling is proposed to be regulated by the core lncRNA TCONS_00028863 via targeting its *cis*-target *RAF43*—analogous to *Brassica rapa* lncRNA *MSTRG.19915*, which forms complementary duplexes with *Brassica rapa MITOGEN-ACTIVATED PROTEIN KINASE 15 (BrMAPK15)* gene to modulate *BrMAPK15* transcription and boost downy mildew resistance [48]. Meanwhile, PP2C5 fine-tunes this process by dephosphorylating key components, preventing excessive activation and balancing defense efficacy with energy costs [49,50]. This regulatory interplay highlights how the pink module integrates metabolic support and precise signal modulation to enable swift PTI responses.

The blue module (MPK3/MPK6-independent PTI and ETI) acts as a “backup network” when the core MPK3/MPK6 pathway is inactivated. Key pathways (innate immunity and photosynthesis) and cytoplasmic transcription factor activity are revealed by functional enrichment of this module, which collectively mediate backup immune responses and immune–growth crosstalk. Bacterial response and stomatal immune signals are integrated by core lncRNAs (e.g., TCONS_00040544) to construct a defense system independent of MPK cascade regulation. A co-expression network is formed between the core lncRNA TCONS_00024195 and its *cis*-target *BHLH48*—a cytoplasmic transcription factor gene. This regulatory pattern likely aligns with a prior study, where resistance to *Xanthomonas oryzae* pv. *oryzae* (*Xoo*, causal agent of bacterial blight) is enhanced by rice lncRNA *ALEX1* via regulating transcription factor gene *AUXIN RESPONSE FACTOR 3 (ARF3)* to activate defense genes [22].

RT-qPCR validation of lncRNAs TCONS_00028863 (targeting *RAF43*) and TCONS_00005620 (targeting *AT1G61640* and *CSU1*) confirmed their elevated expression in WT treated with *Pst* DC3000 *hrcC^−^*, which is consistent with the expression pattern of the “MPK3/MPK6-dependent PTI” pathway (Figure 6). Notably, *RAF43* and the light morphogenesis-related gene *CSU1* have been documented to bridge biotic and abiotic stress responses, facilitating the maintenance of environmental adaptation and immune–growth homeostasis [51]. Signal transduction and cascade-activating proteins, including *AT1G61640* and *RAF43*, act as core components of kinase cascades, relaying upstream signals to activate MPK3/MPK6 and form a pathway-initiating hub [52,53]. These results not only validate the robustness of the transcriptome data but also imply that these lncRNAs may serve as critical regulatory nodes in MPK3/MPK6-mediated rapid defense responses. Specifically, this regulatory role is likely exerted through modulating components of the MAPK cascade (e.g., *RAF43*) and genes associated with light morphogenesis (e.g., *CSU1*).

In summary, this study reveals a multi-layered regulatory framework centered on MPK3/MPK6, with lncRNAs playing a pivotal role in orchestrating adaptive immune responses in plants. These lncRNAs integrate diverse pathogen stimuli and pathway states, coordinating metabolism, signal transduction, and growth regulation through PCGs to provide a robust and flexible immune defense strategy. While the findings highlight the functional significance of lncRNAs in MPK3/MPK6-mediated immunity, further investigation is needed to elucidate the detailed molecular mechanisms underlying their interactions with PCGs. Previous studies have employed CRISPR-Cas9 technology to create lncRNA knockout lines and utilized RNA pull-down assays to verify interaction details, offering valuable insights into these regulatory networks [17]. Additionally, exploring the conservation of these lncRNA regulatory networks in crops could identify new targets for disease resistance breeding, potentially enhancing crop resilience against pathogens [54]. This implies that the lncRNA-PCG pairs identified in this study can be further explored based on these methods to inform strategies for improving plant immunity.

## 4. Materials and Methods

### 4.1. Plant Materials and Treatment

The Columbia ecotype of *A. thaliana* was used in this study, following standard experimental protocols. The *MPK6SR* mutant (harboring *pMPK6::MPK6^YG^* in the *mpk3 mpk6* background) was constructed as previously reported [46,55]. In brief, *MPK6^YG^*—a NA-PP1-sensitive variant of MPK6 driven by its native promoter—was introduced into the *mpk3 mpk6* double mutant to generate the *MPK6SR* line. In the absence of NA-PP1, MPK6^YG^ rescues the embryonic lethality of *mpk3 mpk6*; conversely, treatment with NA-PP1 enables reversible suppression of its kinase activity [46].

*A. thaliana* seeds were surface-sterilized (75% ethanol treatment followed by a rinse with absolute ethanol), dried, and sown on ½-strength Murashige–Skoog (MS) medium. After cold stratification (4 °C, dark, 3 d), seedlings were grown in growth chambers (21 °C, 50% relative humidity, 120 μmol photons·m^−2^·s^−1^, 16 h light photoperiod) for 7 d before being transplanted into soil. For pathogen treatment, three leaves per plant from 4-week-old seedlings were immersed in MES-KCl stomatal buffer (pH 6.15) for 3 h. NA-PP1-pretreated *MPK6SR* and WT plants were then inoculated with one of three solutions: (i) mock control, (ii) *Pst* DC3000 (4 × 10^8^ CFU/mL), or (iii) *Pst* DC3000 *hrcC^−^* (4 × 10^8^ CFU/mL). All bacterial suspensions were prepared in 10 mM MgCl_2_ supplemented with 0.02% Silwet L-77. One hour after treatment, leaves were rinsed with 75% ethanol (10 s), dried, and immediately cryopreserved in liquid nitrogen.

### 4.2. RNA-Seq and lncRNA Identification

High-throughput RNA-seq was performed by NOVOGENE (Beijing, China) using Illumina paired-end sequencing technology. In this experiment, a total of six sample groups were established, including WT treated with mock inoculation (WT-MOCK), inoculated with *Pst* DC3000 (WT-DC3000), or inoculated with *Pst* DC3000 *hrcC^−^* (WT-DC3000 *hrcC^−^*); and *MPK6SR* treated with mock inoculation (*MPK6SR*-MOCK), inoculated with *Pst* DC3000 (*MPK6SR*-DC3000), or inoculated with *Pst* DC3000 *hrcC^−^* (*MPK6SR*-DC3000 *hrcC^−^*). The experiment included three independent biological replicates. For each replicate, one rosette leaf was collected from each of three individual plants. Notably, these three independent replicates were derived from distinct batches of four-week-old *A. thaliana* plants, resulting in a total of 18 samples. This generated 18 raw sequence datasets corresponding to the experimental samples. The raw sequence data reported in this paper have been deposited in the Genome Sequence Archive (Genomics, Proteomics & Bioinformatics 2021) [56] in the National Genomics Data Center (Nucleic Acids Res 2022) [57], China National Center for Bioinformation/Beijing Institute of Genomics, Chinese Academy of Sciences (GSA: CRA028237), which is publicly accessible at https://ngdc.cncb.ac.cn/gsa (accessed on 23 July 2025).

Sequence data processing and genomic mapping were performed as follows: Initial quality control of sequence data was conducted using FastQC (http://www.bioinformatics.babraham.ac.uk/projects/download.html#fastqc; accessed on 20 March 2025) with parameters -q 20 and -p 90 for quality assessment and removal of low-quality reads, followed by adapter trimming and 5′-end nucleotide clipping (7 bp) using the FastX-Toolkit (http://hannonlab.cshl.edu/fastx_toolkit; parameter -f 7; accessed on 21 March 2025). Subsequent genomic alignment involved indexing the reference genome with Bowtie2 (https://sourceforge.net/projects/bowtie-bio/; accessed on 22 March 2025), aligning the processed reads to the indexed genome using TopHat2 (https://ccb.jhu.edu/software/tophat/index.shtml; accessed on 23 March 2025), and performing transcript assembly and isoform-level quantification with Cufflinks (https://mirrors.aliyun.com/macports/packages/cufflinks/; accessed on 26 March 2025) to calculate fragments per kilobase of transcript per million fragments mapped (FPKM) values using the alignment outputs from TopHat2.

Systematic identification of lncRNAs was performed using a multi-stage filtering pipeline [58]: Transcripts with Cufflinks class codes “i” (intronic), “o” (exonic overlap), “j” (novel isoform), “u” (intergenic), or “x” (antisense) were retained in the transcript classification step, followed by discarding transcripts shorter than 200 nucleotides in the length exclusion step; transcripts matching reference mRNAs or structured noncoding RNAs (tRNA/rRNA/snRNA/snoRNA) were eliminated through BLASTN with an E-value < 10^−10^ and sequence identity > 90% in the known RNA filtering step. Subsequently, coding potential assessment was conducted using CPC2, LGC, and Pfam Scan with a stringent threshold (E-value < 10^−5^), retaining only transcripts unanimously classified as noncoding by all three tools; finally, expression thresholding was applied, where final candidate lncRNAs required an FPKM ≥ 0.1 in at least 1 sample. Known lncRNAs also underwent equivalent expression filtering to ensure biological relevance.

### 4.3. Transcriptomic Feature Characterization and Differential Expression Analysis

Bioinformatic analyses were performed using TBtools version 2.221 and GraphPad Prism(version 10), which facilitated comprehensive characterization of *A. thaliana* lncRNA features (including transcript length, GC composition, and exon number) and comparative genomic visualization through Circos plot generation; transcript-level differential expression was assessed using Cuffdiff, with transcripts meeting stringent thresholds (|log_2_FC| ≥ 1, *p* value ≤ 0.05) classified as significantly DElncRNAs or DEPCGs. Intersectional relationships between DElncRNA and DEPCG sets were visualized via Venn diagrams constructed with TBtools, and statistical comparisons of differential expression magnitudes were conducted in GraphPad Prism(version 10) [59].

### 4.4. Identification of lncRNA Targets via Trans-Regulatory Co-Expression Networks

LncRNAs are capable of *trans*-regulating PCGs independent of genomic proximity. To delineate such regulatory interactions, WGCNA was implemented on transcriptomic profiles encompassing both lncRNAs and PCGs. Prior to network construction, low-abundance transcripts were filtered using the median absolute deviation (MAD) criterion, retaining only those with FPKM ≥ 1 in at least 10% of samples; this step yielded 8000 genes for downstream analyses. Key parameters employed for co-expression network construction and module identification included: a scale-free topology fit index (R^2^) threshold of 0.8; a soft-thresholding power of 20; a minimum module size of 90 genes; a maximum block size of 5000 genes; and a module dendrogram cut height of 0.06. Subsequent analyses evaluated associations between the derived expression modules and phenotypic traits of the samples.

To elucidate the biological significance of modules showing significant trait associations, GO enrichment analysis was performed specifically on the PCGs contained within these modules. Within significant modules, highly connected molecules were designated as key regulators: lncRNAs and PCGs that surpassed a predefined weight threshold were classified as hub-lncRNAs and hub-PCGs, respectively. Finally, Cytoscape software version 3.9.1 was employed to generate visual representations of the lncRNA-PCG co-expression networks, thereby illustrating potential *trans*-regulatory interactions [60].

### 4.5. Identification of Cis-Regulated Protein-Coding Gene Targets for lncRNAs

Beyond *trans*-regulatory mechanisms, lncRNAs can also modulate gene expression through *cis*-regulation, which is dependent on genomic proximity. To predict potential *cis*-regulated targets, PCGs located within a 100-kilobase (kb) genomic window flanking each lncRNA locus (both upstream and downstream) were selected [58,59,60,61]. Subsequently, Spearman’s rank correlation coefficients were computed to assess the association between the expression profiles of these spatially adjacent lncRNA-PCG pairs. Pairs demonstrating a strong absolute correlation (|ρ| ≥ 0.9) with statistical significance (*p* value < 0.01) were designated as putative *cis*-regulatory targets. Functional characterization of the identified target PCGs was achieved through GO enrichment analysis. Finally, to depict the co-expression patterns of these candidate lncRNA-PCG pairs, heatmap visualizations were generated using the Heatmap Illustrator module within the TBtools software package, based on FPKM expression values.

### 4.6. RNA Isolation and Quantitative RT-PCR Analysis

RNA extraction was performed on a total of 18 samples from three biological replicates. RNA isolation was performed on leaf tissue from *A. thaliana* plants subjected to low-temperature stress, employing the Eastep^®^ Super Total RNA Extraction Kit (Promega, Madison, WI, USA). Subsequently, 500 ng of purified total RNA per sample underwent reverse transcription to generate complementary DNA (cDNA) using the First-Strand cDNA Synthesis SuperMix kit (TransScript, Beijing, China).

The resultant cDNA was diluted to an appropriate working concentration for RT-qPCR. For each of the 18 samples, three technical replicates (wells) were set up, and the entire experiment was independently repeated three times to ensure reproducibility. Reactions were assembled in a final volume of 20 μL, containing 2 μL of diluted cDNA template combined with 18 μL of master mix incorporating gene-specific primers and SYBR Green detection chemistry (Promega, Madison, WI, USA).

Transcript abundance for target genes was assessed relative to the *ACT2* reference gene [62]. Primer sequences used are listed in Supplementary Table S10. Relative gene expression fold changes were determined using the comparative threshold cycle (2^−ΔΔCt^) method [63]. Statistical analyses evaluating differences between experimental groups were conducted via two-way analysis of variance (ANOVA), followed by relevant post-hoc multiple comparison tests, performed using GraphPad Prism software (version 10) [64].

## 5. Conclusions

The role of lncRNAs in the immunity of *A. thaliana* against *Pst* DC3000 (which induces PTI and ETI) and *Pst* DC3000 *hrcC^−^* (which induces PTI) was investigated in this study, with a focus on their regulation by the MPK3/MPK6 pathway. A total of 1388 known and 70 novel lncRNAs were identified via RNA-seq, and differential expression analysis revealed that lncRNA expression is significantly modulated by MPK3/MPK6—more DElncRNAs were detected in wild-type plants than in *MPK6SR* mutants (which exhibit MPK3/MPK6 deficiency). WGCNA delineated three core modules with distinct functional roles: the “turquoise” module (MPK3/MPK6-dependent PTI and ETI), where lncRNAs integrate metabolic pathways and signal transduction cascades to amplify immune signaling outputs; the “pink” module (MPK3/MPK6-dependent PTI), where lncRNAs mediate rapid defense responses through membrane-localized signaling pathways; and the “blue” module (MPK3/MPK6-independent PTI and ETI), where lncRNAs function as a compensatory regulatory network by modulating cytoplasmic transcription factors and vesicle transport proteins. The *cis*- and *trans*-regulatory analyses uncovered lncRNA-PCG pairs involved in immune signal integration and growth–immunity balance, validated by RT-qPCR for key lncRNAs (e.g., TCONS_00028863), and targets (e.g., *RAF43*). Collectively, these findings establish lncRNAs as critical regulators of plant immunity, acting through both MPK3/MPK6-dependent and -independent pathways. This work not only advances mechanistic understanding of plant immune signaling but also provides valuable molecular resources to inform future research into crop disease resistance.

## Data Availability

The reads are deposited in the National Genomics Data Center (NGDC) under CRA028237.

## Supplementary Materials

The following supporting information can be downloaded at: https://www.mdpi.com/article/10.3390/ijms26178331/s1.
