# Supplementary figures and images for: Transcriptomic Identification of Long Noncoding RNAs Modulating MPK3/MPK6-Centered Immune Networks in Arabidopsis
Source: Int J Mol Sci. 2025 Aug 28;26(17):8331. doi: 10.3390/ijms26178331 (PMC12427685; doi:10.3390/ijms26178331)

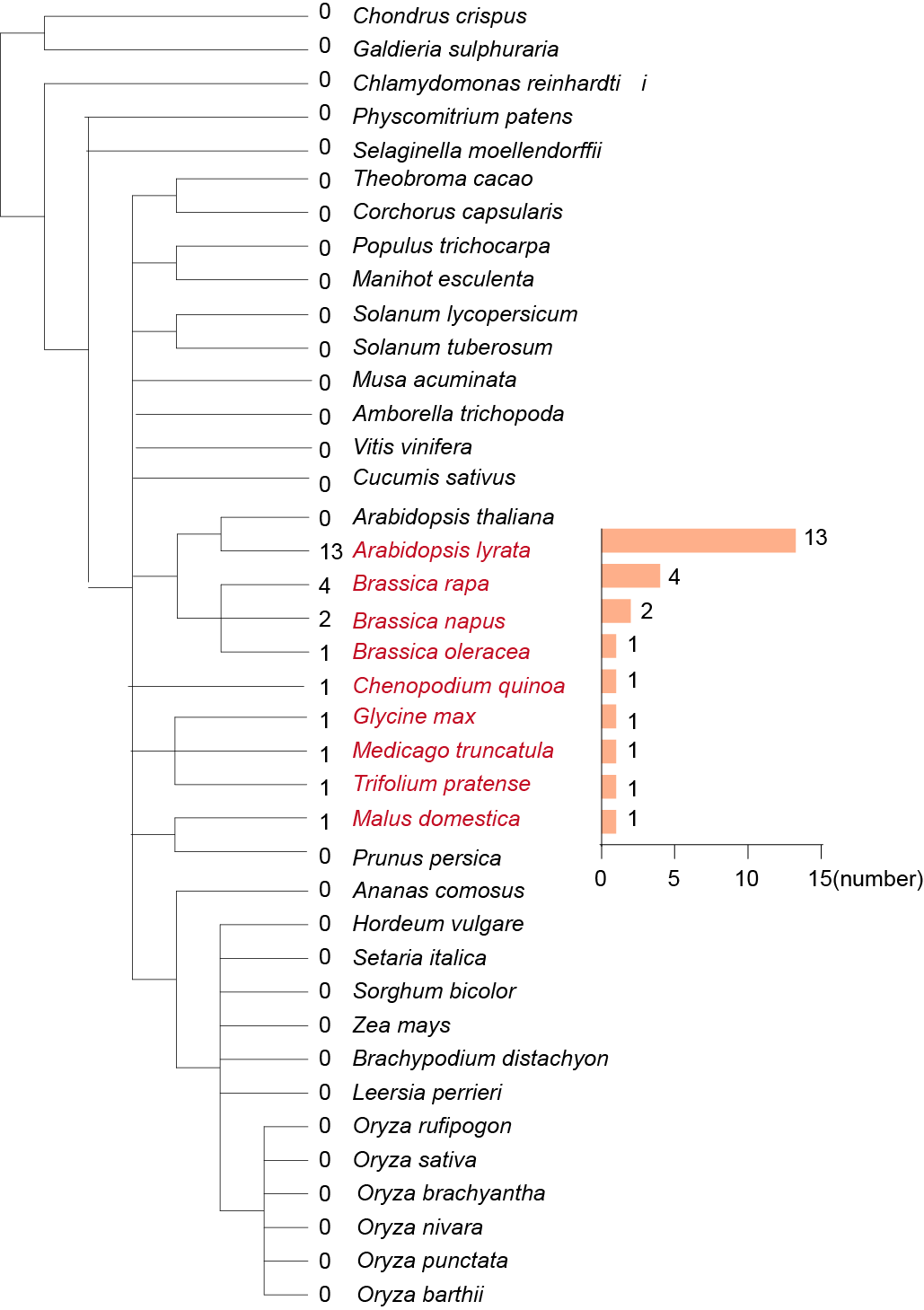

Supplement: Supplementary file 1 [file ijms-26-08331-s001.zip › Figure S2.png]

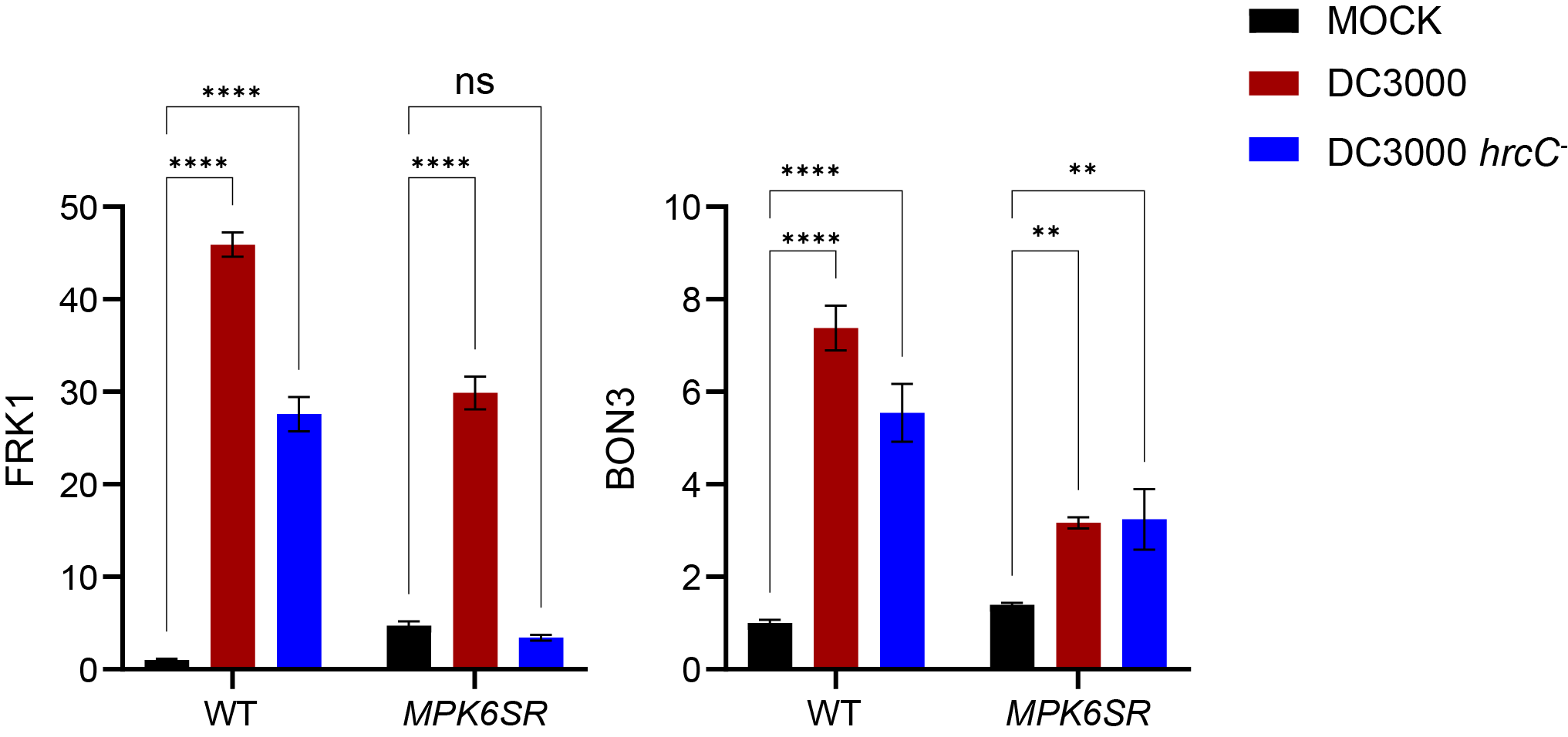

Supplement: Supplementary file 1 [file ijms-26-08331-s001.zip › Figure S1.png]
